# Exclusively extrafacial granuloma faciale: Case report and literature review

**DOI:** 10.1016/j.jdcr.2026.02.014

**Published:** 2026-02-13

**Authors:** Michael J. Ferzoco, Fern A. Wirth

**Affiliations:** a Washington University School of Medicine in St. Louis, St. Louis, Missouri; b Atrius Dermatology, Wellesley, Massachusetts

**Keywords:** case report, chronic inflammatory dermatosis, diagnostic delay, eosinophilic-associated dermatosis, extrafacial granuloma faciale, granuloma faciale, Grenz zone, skin of color

## Introduction

Granuloma faciale (GF) is a rare inflammatory dermatosis of unknown etiology that usually presents as 1 or more persistent erythematous to violaceous plaques on the face of middle-aged Caucasian men.^1^, ^2^, ^3^ Extrafacial lesions are increasingly reported, but under-recognized.^4^^,^^5^ Herein, we describe a young Native American man with long-standing, disseminated exclusively extrafacial GF, accompanied by a literature review that better characterizes the demographic, anatomic, and treatment spectrum of extrafacial GF.

## Case

A 35-year-old Native American man with a past medical history of hyperlipidemia presented to our dermatology clinic with a 9-year history of mildly pruritic erythematous to violaceous plaques on his chest, arms, and back. The patient reported gradual progression of lesions despite treatment with high-potency topical corticosteroids and moderate symptomatic relief from intralesional corticosteroids.

Review of prior records from another dermatology practice revealed that lesions began at age 27 as grouped erythematous papules on the left chest and upper arm without identifiable triggers. Five biopsies over 2 years from multiple sites showed a dense dermal and perivascular lymphocytic infiltrate with neutrophils, eosinophils, plasma cells, and histiocytes, some with periadnexal accentuation and mild mucin deposition; direct immunofluorescence, cluster of differentiation 30, and cluster of differentiation 163 were negative. Histopathological diagnoses ranged from chronic hypersensitivity reaction to atypical granuloma annulare and granuloma annulare–like hypersensitivity reaction. Laboratory testing, including a complete blood count, complete metabolic panel, and serological testing, including Lyme, antinuclear antibodies, double-stranded DNA antibody, and a complement panel, was within normal limits.

**Fig 1:**
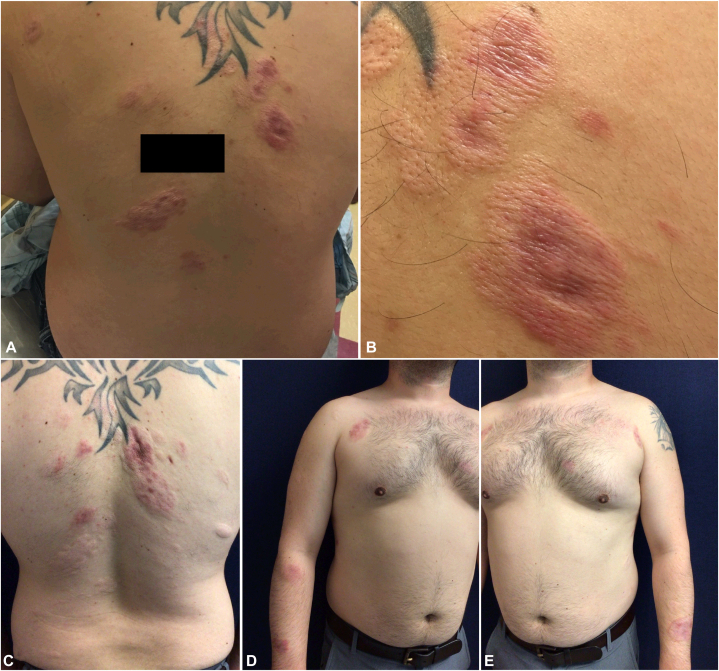
Clinical progression of extrafacial granuloma faciale. **A,** Multiple well-circumscribed, edematous, erythematous to violaceous plaques ranging from several millimeters to several centimeters in diameter on the back. **B,** Close-up of right upper back demonstrating dermal induration, erythema, central darkening, and depression, with accentuation of follicular openings. **C,** Mixed active and atrophic plaques on the back, with post-treatment flattening and pallor following intralesional triamcinolone injections. **D** and **E,** Multiple well-demarcated erythematous to violaceous macules and plaques on the bilateral upper chest and arms.

**Fig 2:**
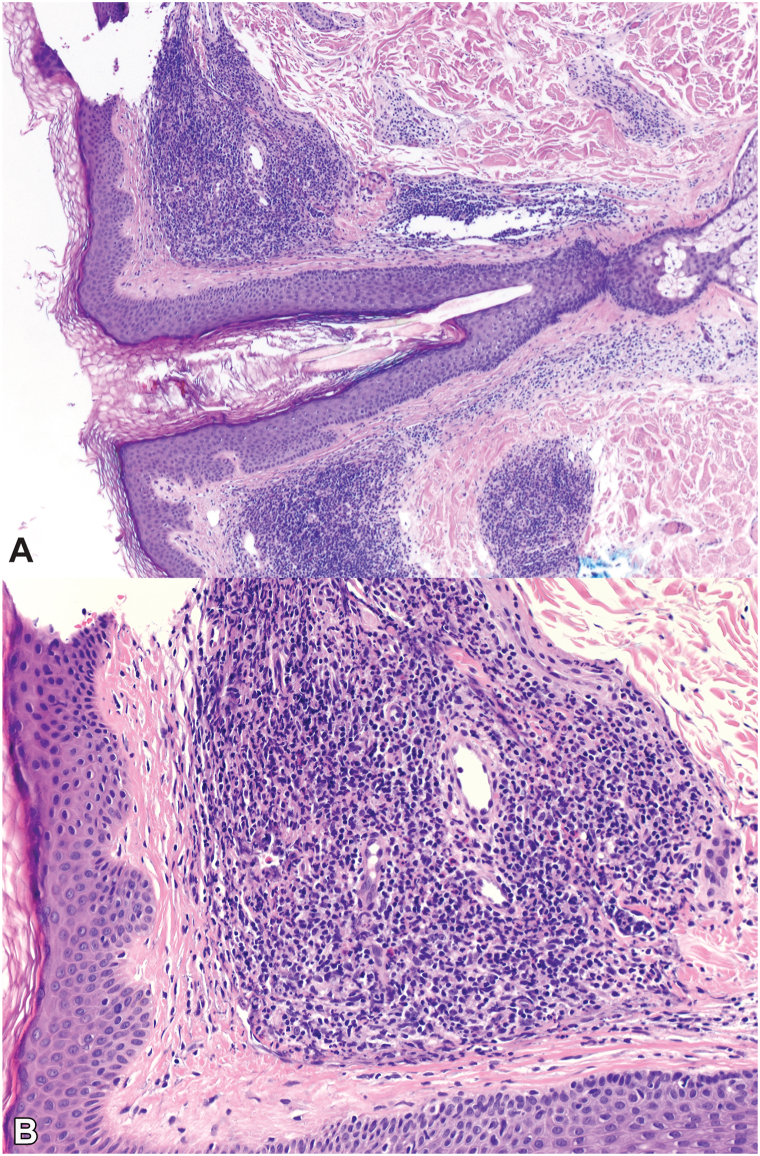
Extrafacial granuloma faciale histopathology. **A,** Dense, mixed, dermal inflammatory infiltrate, with a well-defined Grenz zone, unaffected epidermis, and sparing of adnexal structures (hematoxylin-eosin, 40× magnification). **B,** Polymorphous infiltrate composed of lymphocytes, eosinophils, neutrophils, and plasma cells with leukocytoclasis (hematoxylin-eosin, 200× magnification).

Upon presentation to our practice, many well-demarcated erythematous to violaceous plaques with prominent follicular openings were noted on the chest, back, and arms, without facial involvement (Fig 1, *A* and *B*). A new biopsy from the right upper back was performed and showed a dense, polymorphous dermal infiltrate, with a distinct Grenz zone (Fig 2). No palisading granulomas or mucin deposition were present, and direct immunofluorescence was negative. Repeat laboratory testing for Lyme, rheumatoid factor, erythrocyte sedimentation rate, and antinuclear antibodies remained negative. These findings confirmed a diagnosis of disseminated exclusively extrafacial GF.

Due to suboptimal responses to prior therapy, the patient was started on tacrolimus ointment (0.1%) twice daily, with a moderate reduction in pruritus. Liquid nitrogen cryotherapy was ineffective, and oral hydroxychloroquine, oral dapsone, and 595-nm pulsed dye laser were declined. Intralesional triamcinolone produced the most consistent flattening and decreased erythema, although new lesions continued to develop (Fig 1, *C*-*E*). Throughout the 9-year clinical course, no evidence of facial involvement was seen.

## Discussion

Pathognomonic clinical diagnostic criteria for GF have not been defined.^1^ GF usually presents as an asymptomatic facial papule or plaque; however, extrafacial involvement occurs in 6% to 13% of reported cases.^1^^,^^5^^,^^6^ Our young Native American patient’s lesions were not only exclusively extrafacial but also pruritic, highlighting the variable clinical presentation of GF.^4^

Biopsy remains essential for establishing the diagnosis of GF, although specific histopathological features, such as a Grenz zone, may only be present in 72% to 74% of cases.^1^^,^^2^ Retrospectively, our patient’s initial dense mixed dermal and perivascular infiltrate may have represented an early form of GF prior to the development of the characteristic Grenz zone. GF is increasingly recognized as a misnamed entity, as it is neither granulomatous nor facial in its distinguishing features.

To better characterize extrafacial GF, we performed a structured literature review of all case reports and series on PubMed and Embase containing the terms “granuloma faciale” and “extrafacial” without limitations on language or dates, supplemented by a manual search. A total of 53 articles were identified, containing 81 unique extrafacial GF patients (Supplementary Table I, available via Mendeley at https://data.mendeley.com/datasets/khzwp28m8x/1).

Our review found that the median age at diagnosis of extrafacial GF was 55.4 years, the majority of patients were male (73%), and the mean duration before diagnosis was 59.5 months, which is consistent with published GF data (Table I).^4^^,^^7^, ^8^, ^9^

**Table I:** Clinical features of 81 patients with extrafacial granuloma faciale

Clinical feature	Combined facial/extrafacial (*N* = 44)	Exclusively extrafacial (*N* = 37)	Overall (*N* = 81)
Age, y			
Mean ± SD	51.9 ± 14.1	59.9 ± 14.8	55.4 ± 14.9
Median (IQR)	53.5 (42.2-61.2)	60.0 (51.5-69.5)	57.0 (44.0-67.0)
Range	19-76	28-87	19-87
Sex, no. (%)			
Male	31 (70)	28 (76)	59 (73)
Female	13 (30)	9 (24)	22 (27)
Comorbidity, no. (%)			
None reported	32 (73)	27 (73)	59 (73)
≥1	12 (27)	10 (27)	22 (27)
Extrafacial sites, no. (%)			
1 site	22 (50)	32 (89)	54 (68)
≥2 sites	22 (50)	4 (11)	26 (32)
Duration before diagnosis, mo			
Mean	65.7	50.2	59.5
Range	2.0-480.0	2.0-180.0	2.0-480.0

*IQR*, Interquartile range; *SD*, standard deviation.

In our review, 46% (*n* = 37) of extrafacial GF patients had exclusively extrafacial lesions, representing a higher proportion of this GF subtype than prior reviews (30% to 31%).^5^^,^^6^

Patients with exclusively extrafacial GF were significantly older than those with coexisting facial/extrafacial involvement (median age 60.0 vs 53.5 years; *P* < .05), but these 2 groups did not differ in sex distribution, frequency of reported comorbidities, and duration before diagnosis. Exclusively extrafacial GF was significantly more likely to involve a single anatomic site (*P* < .05), supporting the inclusion of exclusively extrafacial GF in the clinical differential diagnosis of a solitary extrafacial violaceous plaque, particularly in older adults.

The anatomic distribution of extrafacial GF showed a predilection for the upper body and photo-exposed sites, supporting the hypothesis that chronic ultraviolet radiation may be involved in the pathogenesis of GF (Table II).^5^^,^^10^^,^^11^

**Table II:** Anatomic distribution of extrafacial granuloma faciale lesions

Site, no. (%)	Combined facial/extrafacial (*N* = 44)	Exclusively extrafacial (*N* = 37)	Overall (*N* = 81)
Scalp	13 (30)	18 (49)	31 (38)
Back	16 (36)	6 (16)	22 (27)
Shoulders	11 (25)	2 (5)	13 (16)
Arms	7 (16)	2 (5)	9 (11)
Neck	6 (14)	2 (5)	8 (10)
Ears	6 (14)	1 (3)	7 (9)
Chest	6 (14)	0 (0)	6 (7)
Forearms	5 (11)	0 (0)	5 (6)
Abdomen	1 (2)	2 (5)	3 (4)
Genitals	0 (0)	3 (8)	3 (4)
Legs	2 (5)	2 (5)	4 (5)
Trunk	0 (0)	2 (5)	2 (2)
Hands	1 (2)	1 (3)	2 (2)
Axilla	1 (2)	0 (0)	1 (1)
Feet	0 (0)	1 (3)	1 (1)
Tarsal conjunctiva	0 (0)	1 (3)	1 (1)

**Fig 3:**
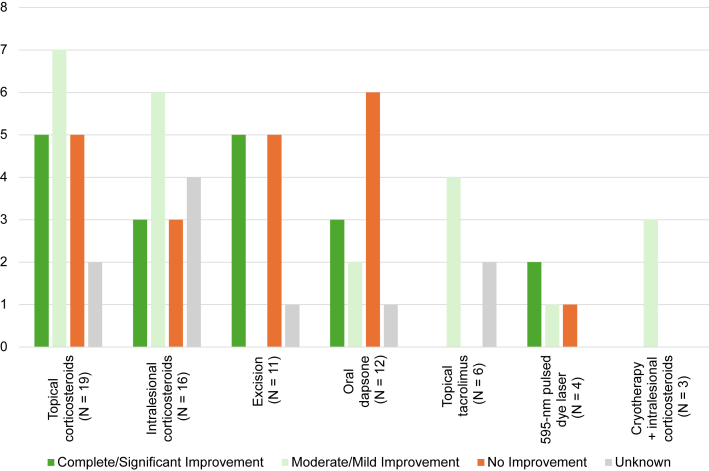
Overview of treatment response for extrafacial granuloma faciale reported in 3 or more patients.

The management of extrafacial GF remains challenging, with variable treatment responses and frequent recurrences.^1^ Similar to a 2018 systematic review of GF, our literature review of extrafacial GF subtypes showed that topical corticosteroids (*n* = 19) and intralesional corticosteroids (*n* = 16) were the most common first-line therapies, with mixed responses (Fig 3).^9^ Both oral dapsone (*n* = 12) and topical tacrolimus (*n* = 6), while moderately effective in that review, showed less consistent benefit in extrafacial GF.^9^ The systematic review also suggested that 595-nm pulsed dye laser is highly effective for treatment-resistant facial GF; however, interpretation in our extrafacial GF cohort is limited by the small number of reported cases (*n* = 4).^9^

Interestingly, surgical excision may be a reasonable treatment for a solitary, exclusively extrafacial GF lesion. Although the sample size was small (*n* = 3), excision of single-site extrafacial lesions resulted in complete clearance without recurrence, highlighting its potential utility in treatment-resistant lesions for this distinct subset.

Due to the rarity of GF, it is likely that case reports will continue to be a primary source of information, and the creation of a multicenter GF registry or shared clinical database could strengthen our understanding of this condition.

Our case of a young Native American man with disseminated exclusively extrafacial GF demonstrates that GF can occur outside traditional demographic boundaries and that clinicians should not exclude GF solely based on age, sex, race, or lesion location(s).

### Declaration of generative AI and AI-assisted technologies in the writing process

During the preparation of this work, the authors used ChatGPT and Google Gemini to support stylistic editing. After using these tools/services, the authors reviewed and edited the content as needed and take full responsibility for the content of the published article.

## Conflicts of interest

None disclosed.

## References

[1] Kang S., Amagai M., Bruckner A.L. (2019).

[2] Ziemer M., Schwede K., Simon J.C., Paasch U. (2013). Atypical erythema elevatum diutinum or extrafacial granuloma faciale?. JDDG - J German Soc Dermatol.

[3] Carlson J.A., LeBoit P.E. (1997). Localized chronic fibrosing vasculitis of the skin: an inflammatory reaction that occurs in settings other than erythema elevatum diutinum and granuloma faciale. Am J Surg Pathol.

[4] Ortonne N., Wechsler J., Bagot M., Grosshans E., Cribier B. (2005). Granuloma faciale: a clinicopathologic study of 66 patients. J Am Acad Dermatol.

[5] Mookadam M., Mesinkovska N., Bridges A.G. (2017). Evaluating the clinical and demographic features of extrafacial granuloma faciale. Cutis.

[6] Deen J., Moloney T.P., Muir J. (2017). Extrafacial granuloma faciale: a case report and brief review. Case Rep Dermatol.

[7] Radin D.A., Mehregan D.R. (2003). Granuloma faciale: distribution of the lesions and review of the literature. Cutis.

[8] Marcoval J., Moreno A., Peyr J. (2004). Granuloma faciale: a clinicopathological study of 11 cases. J Am Acad Dermatol.

[9] Lindhaus C., Elsner P. (2018). Granuloma faciale treatment: a systematic review. Acta Derm Venereol.

[10] Frost F.A., Heenan P.J. (1984). Facial granuloma. Australas J Dermatol.

[11] Rossiello L., Palla M., Aiello F.S., Baroni A., Satriano R.A. (2007). Granuloma faciale with extrafacial lesions. Skinmed.

